# Targeted disruption of the extracellular polymeric network of *Pseudomonas aeruginosa* biofilms by alginate oligosaccharides

**DOI:** 10.1038/s41522-018-0056-3

**Published:** 2018-06-29

**Authors:** Lydia C. Powell, Manon F. Pritchard, Elaine L. Ferguson, Kate A. Powell, Shree U. Patel, Phil D. Rye, Stavroula-Melina Sakellakou, Niklaas J. Buurma, Charles D. Brilliant, Jack M. Copping, Georgina E. Menzies, Paul D. Lewis, Katja E. Hill, David W. Thomas

**Affiliations:** 1 Advanced Therapies Group, Cardiff University School of Dentistry, Heath Park, Cardiff, CF14 4XY UK; 2 AlgiPharma AS, Sandvika, Norway; 3 Physical Organic Chemistry Centre, School of Chemistry, Cardiff University, Cardiff, UK; 4 Respiratory Diagnostics Group, Swansea University, Swansea, UK

## Abstract

Acquisition of a mucoid phenotype by *Pseudomonas* sp. in the lungs of cystic fibrosis (CF) patients, with subsequent over-production of extracellular polymeric substance (EPS), plays an important role in mediating the persistence of multi-drug resistant (MDR) infections. The ability of a low molecular weight (Mn = 3200 g mol^−1^) alginate oligomer (OligoG CF-5/20) to modify biofilm structure of mucoid *Pseudomonas aeruginosa* (NH57388A) was studied in vitro using scanning electron microscopy (SEM), confocal laser scanning microscopy (CLSM) with Texas Red (TxRd®)-labelled OligoG and EPS histochemical staining. Structural changes in treated biofilms were quantified using COMSTAT image-analysis software of CLSM z-stack images, and nanoparticle diffusion. Interactions between the oligomers, Ca^2+^ and DNA were studied using molecular dynamics (MD) simulations, Fourier transform infrared spectroscopy (FTIR) and isothermal titration calorimetry (ITC). Imaging demonstrated that OligoG treatment (≥0.5%) inhibited biofilm formation, revealing a significant reduction in both biomass and biofilm height (*P* < 0.05). TxRd®-labelled oligomers readily diffused into established (24 h) biofilms. OligoG treatment (≥2%) induced alterations in the EPS of established biofilms; significantly reducing the structural quantities of EPS polysaccharides, and extracellular (e)DNA (*P* < 0.05) with a corresponding increase in nanoparticle diffusion (*P* < 0.05) and antibiotic efficacy against established biofilms. ITC demonstrated an absence of rapid complex formation between DNA and OligoG and confirmed the interactions of OligoG with Ca^2+^ evident in FTIR and MD modelling. The ability of OligoG to diffuse into biofilms, potentiate antibiotic activity, disrupt DNA-Ca^2+^-DNA bridges and biofilm EPS matrix highlights its potential for the treatment of biofilm-related infections.

## Drug-resistant biofilms: Marine algal molecules could help

Small carbohydrate molecules derived from marine algae show potential for inhibiting biofilm formation in multi-drug resistant infections. A research team led by Lydia Powell at Cardiff University, UK, investigated the action of carbohydrates called alginate oligosaccharides, composed of a small number of linked sugar molecules. The oligosaccharides modified and disrupted the structure of cultured biofilms of *Pseudomonas aeruginosa*, the cause of many serious drug resistant infections. This effect significantly inhibited the formation and maintenance of the biofilm state, which is known to be a crucial factor allowing the bacteria to resist drug treatment. Antibiotics proved more effective following the oligosaccharide intervention. The researchers uncovered key molecular details involved in the ability of the oligosaccharides to diffuse into and disrupt biofilms. The therapeutic potential of these small carbohydrates is currently being investigated in clinical trials.

## Introduction

Biofilm-associated infections with Gram-negative opportunistic *Pseudomonas* sp. represent a formidable challenge in a range of human diseases, from non-healing skin wounds to chronic respiratory disease. In cystic fibrosis (CF), the colonisation of the lung with *Pseudomonas aeruginosa* is associated with chronic inflammation and deterioration of lung function, leading to a significant increase in morbidity and mortality.^1^ Longitudinal studies in CF have demonstrated that initial pseudomonal colonisation occurs by wild-type (non-mucoid) *P. aeruginosa*. With disease progression and adaptation to the lung environment, *P. aeruginosa* may acquire a mucoid phenotype, with over-production of the exopolysaccharide alginate,^2^ arising predominantly from mutations in *mucA*.^3^

Within biofilm structures, bacteria exhibit increased resistance to conventional antibiotic therapies (up to 10^3^-fold)^4,5^ via a range of indirect and direct mechanisms. These factors include resistance to host-mediated innate and adaptive immune responses,^6^ sequestration of antibiotics in the bacterial periplasm,^7^ reduced bacterial metabolic activity^8^ and development of associated persister cells.^9^ Specific genetic changes may include activation of stress responses e.g., efflux-pumps,^10^ and the density-dependent expression of quorum-sensing (QS) signalling molecules.^11^ These factors confer significant ‘fitness’ advantages for biofilm cells when compared with their planktonic-grown isogenic counterparts.

Although imprecisely defined, bacterial extracellular polymeric substance (EPS) consists mainly of polysaccharides, proteins, lipids and nucleic acids (RNA and extracellular DNA; eDNA) and facilitates biofilm formation and maturation.^12,13^ The effects of eDNA on formation and maturation are mediated, in part, via its direct interaction with calcium (Ca^2+^) within the biofilm, which induces bacterial aggregation via “cationic bridging”.^14^ Extracellular Ca^2+^ also plays an important role within the biofilm in modifying bacterial cell-surface charge, facilitating cellular aggregation and the adherence of bacteria to material/tissue surfaces^15^ by positively charged Ca^2+^ ions overcoming the electrostatic repulsion between negatively-charged biofilm components. *P. aeruginosa* can exhibit distinct differences in the composition of their EPS biofilm matrices; the precise composition being strain-dependent and age-dependent and also strongly influenced by environmental conditions e.g., pH, oxygen tension, and nutrient availability.^12,16^ Studies have previously attempted to define and quantify the EPS component of pseudomonal biofilms^17–20^ and study their distribution in biofilm assembly via selective staining.^20–23^

Non-mucoid *P. aeruginosa* strains produce two important polysaccharides which are involved in biofilm formation namely, Pel (a cationic exopolysaccharide composed of 1–4 linked galactosamine and glucosamine sugars)^24^ and Psl (a penta-saccharide composed of d-glucose, d-mannose and l-rhamnose).^25^ In non-mucoid strains, Psl/Pel predominates in the biofilm matrix, while alginates predominate in biofilms produced by mucoid strains.^26^ The alginates of mucoid *Pseudomonas* sp. are anionic, linear polymers composed of β-d-mannuronic acid (M) and α-l-guluronic acid (G) with a high molecular weight (Mw; 120–480 kDa).^27^ The persistence of pseudomonal infection within the CF lung has, in part, been hypothesised to relate to the emergence of the multi-drug resistant (MDR) mucoid phenotype,^6,28^ with over-production of the alginate exopolysaccharide matrix providing structural integrity to bacterial microcolonies within the diseased lung.^29^ The currently available treatments for pseudomonal infections in the CF lung (via inhaled, oral, or intravenous antibiotic therapies) unsurprisingly have limited efficacy in total biofilm eradication. A large unmet clinical need therefore exists for effective antibiofilm therapies in human disease.^30^

A number of antibiofilm therapeutic strategies have been employed. EPS disruption strategies (including the use of alginate lyase) have, despite advances in enzyme engineering, so far proved ineffective.^31^ More recently, products derived from the marine environment have been investigated for antimicrobial/anti-biofilm activity e.g., furanones from the red algae *Delisea pulchra*.^32,33^ However, none of these potential therapeutic approaches are as yet in clinical use.

Work in our laboratory has demonstrated the ability of a low molecular weight antimicrobial agent, OligoG CF-5/20, (an alginate oligosaccharide derived from the marine algae *Laminaria hyperborea* with a G content >85%), to inhibit Gram-negative bacterial growth and potentiate (synergistically enhance) conventional antibiotics against bacteria in planktonic systems (by up to 512-fold) in the non-mucoid *P. aeruginosa* PAO1.^34^ Recent in vitro studies have also demonstrated the ability of OligoG to potentiate the activity of colistin against mucoid CF isolate *P. aeruginosa* NH53788A^35^ and induce in vivo biofilm disruption in a murine lung infection model.^36^ We hypothesised that the observed potentiation of antibiotics against mucoid *P. aeruginosa* NH57388A was, in part, a direct result of the interaction of OligoG with the EPS component of the bacterial biofilm. To test this hypothesis in vitro, we studied the interaction of OligoG with EPS, and specifically eDNA, in pseudomonal biofilms using fluorescently-labelled oligomers and nanoparticle diffusion to directly quantify these interactions.

## Results

### OligoG inhibits in vitro mucoid biofilm formation

**Fig. 1:**
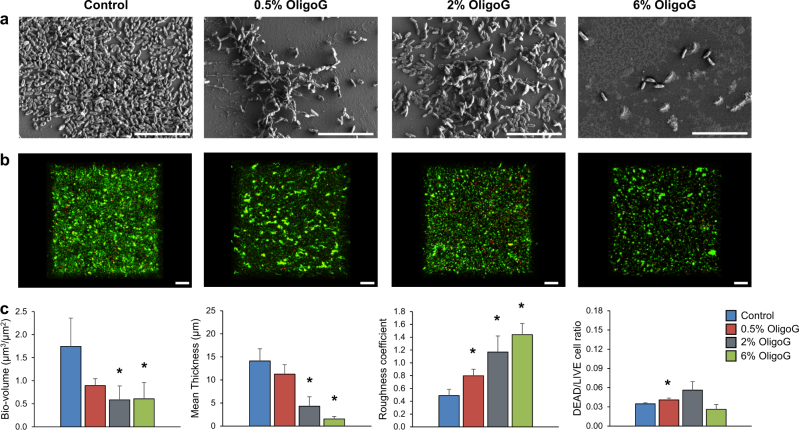
Effect of OligoG on inhibition of mucoid biofilm formation: Imaging and quantification of *P. aeruginosa* (NH57388A) biofilms grown for 24 h at 37 °C in MH broth ± OligoG (0.5%, 2% & 6%). **a** SEM imaging of biofilms (Scale bar, 10 µm). **b** CLSM 3D imaging (aerial view) of LIVE/DEAD® staining of biofilms (Scale bar, 20 µm). **c** COMSTAT image analysis of the corresponding biofilm CLSM z-stack images. *P < 0.05 significance was determined by comparison to the untreated control. Error bars represent the standard deviation of the data set (*n* = 3)

Scanning electron microscopy (SEM) imaging demonstrated a dose-dependent reduction in *P. aeruginosa* NH57388A bacterial density and biofilm growth in the presence of OligoG (Fig. 1a). This biofilm inhibition was quantified and confirmed with COMSTAT analysis of confocal laser scanning microscopy (CLSM) z-stack images, showing that OligoG (≥2%) significantly disrupted biofilm growth in a dose-dependent manner, exemplified by decreased mean biofilm bio-volume and thickness and increased roughness coefficient following treatment (Fig. 1b, c; *P* *<* 0.05). CLSM imaging also revealed that the presence of OligoG was associated with dose-dependent bacterial aggregation, which was particularly evident at concentrations ≥0.5%.

### OligoG disrupts established in vitro mucoid biofilms

**Fig. 2:**
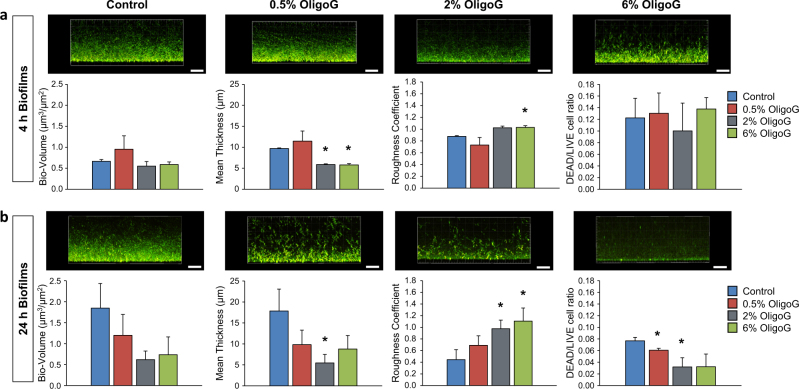
Effect of OligoG on disruption of mucoid established biofilms: CLSM 3D imaging (side view) with LIVE/DEAD® staining of *P. aeruginosa* (NH57388A) biofilms grown for 24 h at 37 °C in MH broth followed by 4 or 24 h OligoG treatment (0.5, 2 & 6%) (Scale bar, 15 µm) with COMSTAT image analysis of the corresponding biofilm CLSM z-stack images. Treatment times **a** 4 h. **b** 24 h. *P < 0.05 significance was determined by comparison to the untreated control. Error bars represent the standard deviation of the data set (*n* = 3)

Growth of *P. aeruginosa* NH57388A for 24 h generated well-defined 3-dimensional biofilm structures (mean thickness of 17.8 ± 5.2 µm). CLSM imaging and COMSTAT analysis revealed that the effect of OligoG on these established biofilms was time-dependent (Fig. 2). Although treatment for 1 h appeared to have no effect on biofilm structure (Fig. S1), 4 h treated biofilms showed a dose-dependent reduction in biofilm thickness at OligoG concentrations ≥2%, and a significant increase in roughness coefficient at OligoG concentrations ≥6% (*P* *<* 0.05; Fig. 2a). After 24 h treatment there was a more marked reduction in biofilm bio-volume and a significantly decreased biofilm thickness with increased OligoG concentrations (*P* *<* 0.05; Fig. 2b). These effects were also dose-dependent, with significantly increased roughness coefficients and a decrease in the numbers of non-viable bacterial cells observed with increasing OligoG concentration in the treated samples (*P* *<* 0.05; Fig. 2b).

### Texas red (TxRd®) labelling studies demonstrate diffusion of the oligomer into mucoid biofilms

OligoG was labelled with Texas red (TxRd®) to facilitate visualisation of OligoG within the biofilms (Fig. S2a, b, c). CLSM images of OligoG (≥0.5%) treated *P. aeruginosa* (NH57388A) biofilm formation (24 h) (Fig. S3a) and biofilm disruption (Fig. S3b) showed TxRd®-associated fluorescence was distributed throughout the whole biofilm structure. Furthermore, CLSM images emphasised the biofilm inhibitory and disruptive properties of TxRd®-OligoG, displayed by a significant dose-dependent decrease in mean biofilm bio-volume and increase in roughness coefficient with increasing TxRd®-OligoG concentrations for both biofilm formation (Fig. S4a) and biofilm disruption (Fig. S4b) assays (*P* *<* 0.05).

**Fig. 3:**
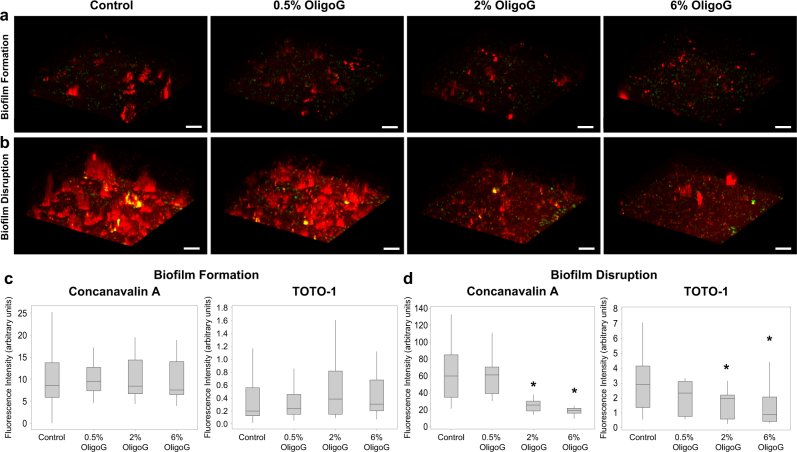
Effect of OligoG on the EPS components of mucoid biofilms. 3D CLSM imaging of *P. aeruginosa* (NH57388A) biofilms stained with ConA (EPS polysaccharides, red) and TOTO-1 (eDNA, green; scale bar, 8 µm) in **a** biofilm formation assay, where biofilms are grown for 24 h in MH broth ± OligoG (0.5%, 2% & 6%) and **b** the biofilm disruption model where biofilms grown for 24 h in MH broth, followed by 24 h treatment of OligoG (0.5%, 2% & 6%). Corresponding mean fluorescence intensities (arbitrary units ×10^6^) achieved from CLSM 3D imaging of the **c** biofilm formation assay and the **d** biofilm disruption assay. *P < 0.05 significance was determined by comparison to the untreated control. Error bars represent the standard deviation of the data set (*n* = 3)

Although un-conjugated “free” TxRd® dye was shown to penetrate to the base of the biofilm, this was not associated with alteration of biofilm architecture or bacterial aggregation (see Fig. S4c). In contrast, penetration of the labelled OligoG to the base of established biofilms was associated with bacterial aggregation (Fig. S2c). Analysis of the supernatant by size exclusion chromatography demonstrated that there was no detectable free TxRd® present, thereby confirming that TxRd® was not released from OligoG during the experiment (Fig. S2b; TxRd® eluted at >5.5 ml).

### OligoG does not cause an increase in planktonic cell numbers after treatment of established biofilms

To determine the effect of the oligomers on possible dispersal of planktonic cells following treatment of established (24 h) biofilms, analysis of the cell supernatant was performed. This revealed that the observed decrease in biofilm bio-volume and thickness (Fig. 2b) was not directly related to increased numbers of planktonic cells in the growth medium following 24 h OligoG treatment. Instead, there was a dose-dependent decrease in planktonic cells that was significant at 6% OligoG (*P* *<* 0.05; see Fig. S5), presumably as a direct result of reduced growth following treatment.

### OligoG disrupts EPS within the mucoid biofilm matrix

*P. aeruginosa* NH57388A biofilms were stained to visualise different components of the biofilm using SYPRO® Ruby Red (proteins; Fig. S6), Alexa Fluor 633-labelled Concanavalin A (α-d-glucose and α-d-mannose; ConA) and TOTO-1 (eDNA) (Fig. 3a, b).^20,21^ SYPRO® Ruby Red staining demonstrated a dose-dependent inhibition of biofilm matrix formation (specifically the protein component of the biofilm) with increasing OligoG concentration (see Fig. S6). However, this same trend was not observed in fluorescence intensities quantified from CLSM images achieved using ConA and TOTO-1 staining (Fig. 3a), which revealed no significant changes in fluorescence intensity (for either polysaccharides or eDNA respectively) during biofilm formation following OligoG treatment (Fig. 3c). Instead, from the CLSM images of biofilm formation, there appeared to be a change in the distribution of the ConA staining following OligoG treatment (>0.5%), with a trend towards smaller clusters of ConA-stained EPS components (polysaccharides) (Fig. 3a). In contrast, CLSM images (Fig. 3b) and corresponding fluorescence intensity measurements of *P. aeruginosa* NH57388A 24 h established biofilms treated with OligoG, revealed a significant dose-dependent reduction in the fluorescence intensity of both ConA and TOTO-1 staining following treatment (*P* *<* 0.05), indicating biofilm disruption through reduction in both the polysaccharides and eDNA components of the biofilm matrix (Fig. 3d).

### OligoG treatment increases nanoparticle diffusion within biofilms

**Fig. 4:**
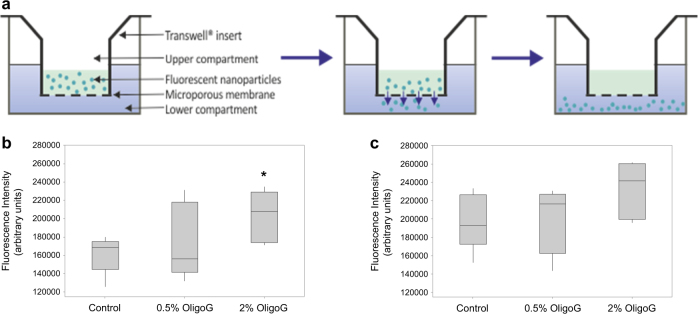
Transwell® biofilm diffusion studies. **a** Schematic diagram of Transwell® device showing particle diffusion through the biofilm and microporous membrane. Boxplots of mean fluorescence intensity (arbitrary units) in the biofilm Transwell® assay after 4 h OligoG treatment (n = 5), where fluorescence was measured at **b** 1 h and **c** 2 h after fluosphere addition. *P < 0.05 significance was determined by comparison to the untreated control. Error bars represent the standard deviation of the data set (*n* = 3)

A Transwell® biofilm diffusion assay was developed employing fluorescent nanoparticles to measure particle diffusion through biofilms, treated with/without OligoG for 4 h (Fig. 4a). OligoG treatment (2%) of biofilms increased the diffusion of fluorescent nanoparticles at 1 h and 2 h incubation after nanoparticle addition (Fig. 4b, c), which was significantly different at the 1 h time point (*P* < 0.05).

### OligoG acts synergistically with the antibiotic erythromycin and tobramycin against biofilms

Minimum inhibitory concentration (MIC) assays and CLSM imaging were performed to visualise the potential synergy between OligoG and the antibiotics erythromycin and tobramycin. The MIC for erythromycin was reduced four-fold against the *P. aeruginosa* NH57388A, from 128 μg ml^−1^ (0% OligoG) to 32 μg ml^−1^ after treatment with 6% OligoG, whilst the MIC for tobramycin against *P. aeruginosa* NH57388A remained unchanged with OligoG treatment (1 μg ml^−1^). CLSM images also showed increased biofilm disruption and increased numbers of non-viable cells in biofilms treated with 2% OligoG and 128 μg ml^−1^ (MIC value) erythromycin, in contrast to the erythromycin-only treated control (see Fig. S7). In biofilms treated with 2% OligoG and 1 μg ml^−1^ (MIC value) tobramycin whilst disruption of the treated biofilms was also evident, no similar increase in non-viable cells was observed (Fig. S7).

### Molecular dynamics (MD) simulations demonstrated contrasting interactions of OligoG with calcium and DNA

**Fig. 5:**
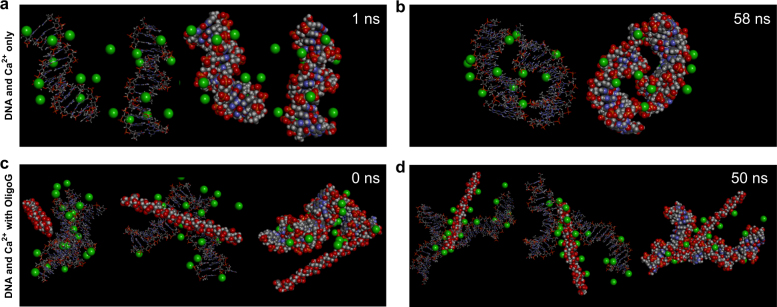
Molecular dynamics (MD) simulations of calcium, DNA and OligoG interactions. MD simulations at early and late binding of DNA double strand (15 bp) in the presence of Ca^2+^ (green spheres) at **a** 1 ns and **b** 58 ns. G-oligomer (DPn 16) was added to the simulations where the binding affinity was assessed at **c** 0 ns and **d** 50 ns

MD modelling demonstrated the ability of Ca^2+^ ions to rapidly bind pseudomonal DNA (in <1 ns; Fig. 5a), illustrating clearly how Ca^2+^-DNA-Ca^2+^ “bridges” may be formed in vivo (between individual DNA molecules in ~ 60 ns; Fig. 5b). Ca^2+^ binding appeared to initiate the formation of these bridges, and once formed, the bond remained stable, while further bridging occurred. Addition of OligoG (DPn 16) in these models (Fig. 5c), demonstrated its interaction with the Ca^2+^ ions both present in the Ca^2+^-DNA-Ca^2+^ “bridges” and its rapid sequestration of free Ca^2+^ ions. The simulation revealed the ability of the oligosaccharide to invade the structural assembly and disrupt these existing bridges between adjacent DNA molecules, resulting in the formation of stable DNA-Ca^2+^-OligoG-Ca^2+^-DNA bridged complexes (Fig. 5d). The simulations, in which “free Ca^2+^” ions were removed, revealed the limited ability of OligoG to remove Ca^2+^ molecules already bound to DNA.

### OligoG exhibits contrasting molecular interactions with calcium and DNA

**Fig. 6:**
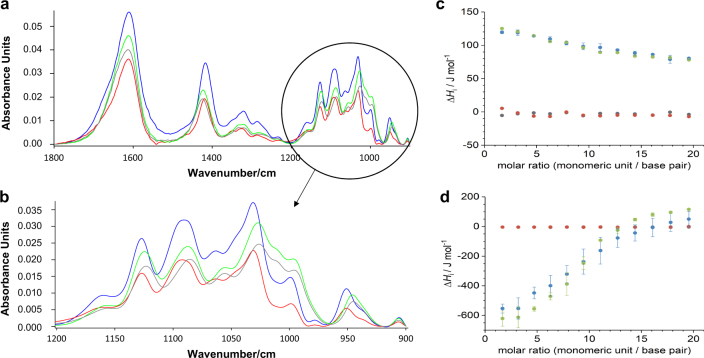
FTIR and ITC analysis of interactions of OligoG with DNA and Ca^2+^. FTIR absorbance spectra of the molecular interaction of OligoG with calcium and DNA **a** from 1800 to 900 cm^−1^ of pure OligoG (blue), OligoG + 5 mM Ca^2+^ (green), OligoG + 10 mM DNA + 5 mM Ca^2+^ (grey), and OligoG + 10 mM DNA (red) and **b** from 1200–900 cm^−1^ (colours as in A). Isothermic calorimetric titrations of 101 mM OligoG into 1 mM fish sperm (FS) DNA containing **c** 1 mM CaCl_2_ or **d** 5 mM CaCl_2_ (and corresponding reference experiments); buffer into buffer (grey), buffer into DNA (red), OligoG into buffer (blue), OligoG into FS DNA (green). Concentrations of OligoG and FS DNA are in terms of monomeric units and base pairs, respectively. Error bars present the standard deviations of the data set (*n* = 3)

The interaction of the alginate oligosaccharide, OligoG, with Ca^2+^ ions was also readily demonstrated by fourier transform infrared spectroscopy (FTIR; Fig. 6a) being evident in peak-shift, most markedly in the “glycogen-rich” region of the spectrum (1200–900 cm^−1^; Fig. 6b and Fig S8a). The lack of novel peaks suggested that Ca^2+^ ions interact with OligoG via specific molecular interactions, via electrostatic and/or ionic interactions, influencing the stretching/bending moments of the C–O bonds characteristic of this region. By contrast, FTIR spectral analysis failed to demonstrate any major differences in the OligoG spectra following DNA treatment, with little change in peak positions (Fig. 6b).

### OligoG does not show instantaneous interactions with DNA

The interactions of OligoG with DNA in the presence of 1 and 5 mM Ca^2+^ were studied using isothermal titration calorimetry (ITC). The heat effects for dilution of OligoG in the presence of Ca^2+^ were not constant, indicating self-aggregation of OligoG. In the presence of 1 mM Ca^2+^, the observed dilution heat effects were endothermic (Fig. 6c) whereas they were exothermic in the presence of 5 mM Ca^2+^ (Fig. 6d). In the presence of 5 mM Ca^2+^ the dilution heat effects showed stronger variation than in the presence of 1 mM Ca^2+^, suggestive of synergy in the process of calcium binding to alginates at higher Ca^2+^ concentrations. Comparison of the molar heat effects for dilution of OligoG into buffer with the molar heat effects for titration of OligoG into DNA however, showed no significant differences. Hence, in the presence of 1–5 mM Ca^2+^, interactions between DNA and OligoG were either too weak or occurred on too long a timescale (longer than tens of seconds) to be detected using ITC. Increasing the DNA concentration to 10 mM DNA in the presence of both 1 and 5 mM Ca^2+^ demonstrated the same trend in the presence of OligoG (Fig. S8b and c).

## Discussion

Bacterial biofilms represent a considerable challenge for antimicrobial therapy. Therefore, in the development of novel therapeutic approaches, it is important to determine the extent to which such new drugs can penetrate the biofilm and disrupt the components of the EPS.^37^ The observed inhibition of pseudomonal biofilm formation in the presence of OligoG is in accord with previous in vitro AFM force-measurement and rheological studies, where OligoG induced discrete mechanical alterations within biofilms.^38^ Also, a recent in vivo murine lung infection model study^36^ confirmed that OligoG disrupted biofilm formation in a dose-dependent manner. The mechanisms by which these changes may occur in developing biofilms, may relate to several previously-described effects of the alginate on planktonic *P. aeruginosa* PAO1 including: binding to the Gram-negative bacterial cell surface, forming microbial aggregate complexes with OligoG,^39^ inhibition of growth,^34^ decreased bacterial surface attachment,^40^ modulation of surface charge (zeta potential) and reduction of bacterial motility.^39^ All of these effects could readily modify biofilm formation. However, these mechanisms would not affect the marked disruption evident in the dense (approximately 20 μm thick), established (24 h) NH57388A biofilms. The dose-dependent disruption of established biofilms with oligomer treatment was however, clearly evident from the COMSTAT analysis;^41^ characterised by marked decreases in biofilm bio-volume (up to 3-fold reduction) and mean thickness.

A potential mechanism by which anionic low Mw oligomers might act in established biofilms is via disruption of the EPS components of the biofilm matrix.^42^ We hypothesised that gross, dose-dependent biofilm disruption would necessitate the effective diffusion of OligoG throughout the whole biofilm matrix. The use of labelled oligomers allowed us to study the ability of OligoG to diffuse into the complex, “branching” mesh of the hydrated biofilm EPS. TxRd®-labelled OligoG could be seen throughout the biofilm network, both during biofilm formation (formed in the presence of treatment) at OligoG concentrations as low as 0.5%, and also after treatment of established biofilms, demonstrating effective diffusion of the negatively-charged oligomers into the biofilm structure and their ability to induce bacterial aggregation (in contrast to the TxRd® control). This study confirmed that the efficacy of OligoG was not biofilm surface-restricted and moreover, that TxRd®-labelling did not impair the anti-biofilm effects of OligoG.

The importance of eDNA in pseudomonal biofilm architecture has been demonstrated in directing both cell-surface attachment^43,44^ and providing mechanical stability.^14^ Within the diseased lung, eDNA may be derived from autolysis of the biofilm bacteria and host-derived immune cells.^21^ No apparent difference in the quantity of eDNA was observed in biofilms formed in the presence of OligoG. Therefore, the ability of OligoG to inhibit biofilm formation may be related to the previously described activity of the oligomers on planktonic Gram-negative bacteria, such as *P. aeruginosa* PAO1, where treatment modified bacterial growth,^34^ cell-surface charge,^39^ and inhibited bacterial attachment.^40^ In contrast, imaging studies of established biofilms demonstrated the ability of OligoG (≥2%) to significantly reduce biofilm eDNA. Interestingly, the previously demonstrated ability of the oligomers to modify *pilE* gene expression and swarming motility in *P. aeruginosa*,^39^ and induce dose-dependent reductions (>0.2%) in non-mucoid pseudomonal QS signalling^35,45^ suggests that modulation of QS signalling within the biofilm environment may also play a direct role in mediating the observed changes in biofilm architecture and eDNA.

Lectin staining combined with CLSM imaging provided not only a visual demonstration of the effects of OligoG on the EPS biofilm matrix, but also enabled quantification of the structural changes in the biofilm matrix. ConA staining with CLSM imaging demonstrated that the oligomers morphologically altered the distribution of EPS polysaccharides during biofilm formation and also following treatment of established biofilms; fluorescence intensity measurements revealed significantly decreased polysaccharides in the oligomer-treated established biofilms. The glucose-rich polysaccharide Pel (a cationic EPS component produced during *P. aeruginosa* biofilm formation) is involved in cell-to-cell interactions,^17^ and has recently been demonstrated to ionically cross-link eDNA within the biofilm matrix,^24^ whilst the mannose-rich Psl (a key constituent of biofilm scaffolding) mediates both cell-surface and cell–cell interactions.^18–20^ Kundukad et al.^46^ recently demonstrated that differences in Psl concentration (occurring with biofilm maturation) resulted in altered mechanical properties of biofilms. Moreover, it has been hypothesised that pseudomonal rugose small-colony variants, expressing increased levels of Pel and Psl, might play an important role in mediating bacterial persistence following prolonged antibiotic treatment, within the CF lung.^47^ Pseudomonal alginates are also key components of the biofilm matrix in the diseased CF lung and contribute to the thick, viscous mucus.^48^ Mucoid strains of *P. aeruginosa* typically produce high-Mw (>15 kDa) naturally acetylated alginates^13^ which lack consecutive G-residues and contribute to the mechanical strength of the biofilm, via chelation of divalent cations (e.g., Ca^2+^ and Mg^2+^).^49^ Due to the structural importance of the EPS in maintaining biofilm physiology, numerous strategies have been developed in an attempt to effect EPS disruption including the use of alginate lyase, DNase and hydrolase-based approaches.^30,50,51^ OligoG may also modify the structure of EPS via its ability to bind divalent cations, e.g., Ca^2+^ and Mg^2+^, which are involved in regulating EPS and eDNA interactions within the biofilm scaffold;^43,48^ Ca^2+^ also appearing to play an important role in the switch in *P. aeruginosa* from acute to chronic virulence.^52^ Chelation by OligoG results from the Ca^2+^-binding affinity of the conformational arrangement of the G-block co-polymer. Alginates, such as OligoG, are known to interact with Ca^2+^ in a dose-dependent process, displaying auto-cooperativity^53–56^ and the recently observed ability of OligoG to modify the assembly of the dense CF intestinal mucus has been hypothesised to occur via chelation of Ca^2+^.^57–59^ Bacterial polysaccharides, Ca^2+^ and eDNA are key, inter-related components of biofilms which direct assembly, architecture and resistance to therapy.^14,58^ Disruption of these important biofilm matrix components by the negatively-charged OligoG could have contributed to the observed changes in bio-volume and biofilm thickness in both biofilm formation and treated established biofilms. It is important to note however, that ConA binding is not solely restricted to α-d-glucose and α-d-mannose; Con A may also label high-molecular weight bacterial-derived alginate block co-polymers, within the biofilm structure.^22^

Previously, Ca^2+^-mediated interactions between the lipopolysaccharides (LPS) of Gram-negative bacteria and OligoG^60^ and DNA^61^ have been demonstrated. Instead here, we studied the potential interactions between OligoG and DNA mediated by Ca^2+^ that could occur within pseudomonal biofilms. MD simulations suggested that OligoG might effectively disrupt the assembly of new DNA-Ca^2+^-DNA bridges in biofilm development, in keeping with the CLSM studies. The ability of OligoG to “invade” these established DNA-Ca^2+^-DNA networks was supported by the effective diffusion of fluorescently-labelled oligomers into 24 h biofilms. This proposed invasion/disruption may be reflected in the previously altered viscoelastic properties of OligoG-treated pseudomonal biofilms.^38^ Furthermore, the MD simulations suggested that, once bound, the “free end” of the oligomer retained the ability to bind free-Ca^2+^ (and adjacent OligoG molecules). This finding may explain the dose-dependent aggregation in the treated samples observed here, and in previous studies.^39^ The ITC experiments (at higher Ca^2+^ concentrations) also reflected this Ca^2+-^dependent, self-aggregation of OligoG.

The MD simulations suggested the importance of the interactions with Ca^2+^, rather than direct interactions of the OligoG with the DNA in our biofilm models. It must be noted however, that the simulation was designed to study Ca^2+^-DNA interactions, (and other divalent cations e.g., Mg^2+^ which are also bound by the oligosaccharides) which may be modified in vivo. FTIR showed a lack of apparent molecular interaction between OligoG and DNA. Furthermore, ITC demonstrated that, even in the presence of calcium, OligoG and DNA do not interact instantaneously, suggesting that the biofilm-disrupting effects of OligoG are not the result of a (rapid) Ca^2+^ mediated interaction between OligoG and eDNA and would not induce immediate biofilm destabilisation. The lack of instantaneous interactions between OligoG and DNA is in agreement with the observed time-dependent disruption of treated established biofilms. Whilst all of these in vitro experiments do not reproduce the complex relationship that exists between eDNA, Ca^2+^ and bacterial polysaccharides within biofilms in vivo, they are useful models to study potential interactions.

Elevated levels of cations (up to 120 mg l^−1^ Ca^2+^) are evident locally in the diseased lungs of CF patients and have been hypothesised to contribute to the severity of disease.^62^ Several studies have consequently considered the possible therapeutic utility of chelating agents to enhance biofilm disruption and facilitate antibiotic potentiation in vitro.^63^ In contrast to “broad-spectrum” chelating agents such as EDTA, OligoG lacks toxicity issues. Hence in terms of CF treatment, the effect of OligoG on both divalent cations and eDNA is highly beneficial for these patients, in whom pulmonary levels of Ca^2+^ and Mg^2+^ may be elevated.

OligoG has been proven to be safe for inhalation therapy and has been shown to alter the viscoelasticity of CF sputum.^64^ OligoG has been demonstrated to not only reduce bacterial load within biofilm structures, but also to target and disrupt the EPS matrix of established biofilm architecture. Conceptually, this disruption of established biofilms may facilitate penetration of the biofilm-coated lung surface, which is an effective barrier to the delivery of pharmacotherapy and gene-therapy across the lung-surface in disease.^37^ To test this, we utilised a fluorescent, particle-diffusion model using biofilms in a Transwell® system, which has recently been used to study fluorescent particle permeation through mucus.^65^ Negatively-charged particles were employed since the EPS matrix of biofilms also has an overall negative net charge^66^ and a number of negatively-charged antibiotics may also be used in the treatment of Gram-negative biofilm infections e.g., ciprofloxacin.^25,67^ The Transwell® assay, developed here to model diffusion through bacterial biofilms, has the advantage of measuring the ‘bulk’ properties of the entire biofilm. In this model, it was clear that the dose-dependent increase in biofilm disruption and decreased thickness evident from the CLSM imaging was also reflected in increased particle diffusion. Although significantly increased diffusion was observed in the Transwell® assay, the standard deviations were relatively high, indicating possible pore-blockage^68^ and/or the non-uniformity of biofilm disruption observed in the CLSM imaging. These experiments used oligomer concentrations of ≤2% to facilitate rinsing of the biofilms. Despite these issues, the results revealed the utility of the Transwell® particle diffusion assay to model the efficacy of anti-biofilm strategies.

Baker et al.^69^ recently demonstrated that the biofilm EPS-disrupting ability of glycoside hydrolases (PelA_h_ and PslG_h_) was accompanied by potentiation of the antibiotic colistin. Workers have recently demonstrated ability of OligoG to synergistically enhance the activity of colistin against *P. aeruginosa* NH57388A in minimum biofilm eradication concentration assays.^36^ We showed here that OligoG also potentiated the antibiofilm activity of the macrolide antibiotics, erythromycin and tobramycin against *P. aeruginosa* NH57388A biofilms. The results were contrasting, and not as marked in tobramycin, which may reflect, in part, the interaction of the anionic OligoG, with the polycationic tobramycin. Interestingly, in addition to disruption of the EPS, the ability to potentiate antibiotic activity against biofilms described here may also be mediated via the ability of OligoG to modify pseudomonal motility,^34^ where Chua et al.^70^ recently demonstrated the importance of motility in the acquisition of colistin resistance within pseudomonal biofilm communities.

These studies demonstrate the effects of the inhaled alginate oligomer therapy, OligoG, in impairing the formation and facilitating disruption of mucoid biofilms. Whilst the mechanisms of disruption of biofilm EPS matrix and the modification of eDNA assembly in the biofilms may be regulated indirectly (via QS signalling) these studies demonstrate the mechanistic importance of direct interaction of OligoG with Ca^2+^ and the resultant modification of intercellular bridges within the Ca^2+^- DNA biofilm mesh.

The direct and indirect anti-biofilm effects, coupled with the safety and tolerability of OligoG CF-5/20 may have potential clinical utility in the management of biofilm-related Gram-negative infections. Phase IIb clinical studies are ongoing in CF patients (www.ClinicalTrials.gov [NCT02157922], [NCT02453789]).

## Materials and methods

### Alginate oligosaccharide synthesis (OligoG)

OligoG CF-5/20 was produced from the stem of the brown seaweed *Laminaria hyperborea* and purified and fractionated as previously described.^32^ This resulted in a low molecular weight (mean Mn 3200 g mol^−1^) alginate oligomer possessing a high guluronate content (>85%) with a degree of polymerisation [DPn] of 16.

### Bacterial strains and media

A mucoid, CF *P. aeruginosa* clinical isolate (NH57388A) was used in this study.^71–73^ Bacterial colonies were grown on blood agar no.2 (BA; Lab M) supplemented with 5% horse blood. Overnight cultures were grown in tryptone soya broth (TSB; Lab M) at 37 °C, with shaking. Cultures were adjusted to 10^7^ cfu ml^−1^ (~OD_600_ 0.05)^74^ before use in the following experiments.

### Effect of OligoG as a treatment to inhibit biofilm formation (SEM)

Mueller Hinton (MH) broth (Lab M) ± 0.5%, 2% or 6% OligoG (w/v) was prepared in a 12-well plate (Greiner Bio-One, Stonehouse, UK) containing Thermanox^TM^ slides (Agar Scientific), followed by an inoculum using a 1:100 dilution of the *P. aeruginosa* (NH57388A) overnight culture, and incubated at 37 °C with gentle rocking for 24 h. The supernatant was then removed and biofilms fixed with 2.5% (v/v) glutaraldehyde for 1.5 h. Following washing (x4) with dH_2_O, the fixed biofilms were covered with 1 ml dH_2_O, frozen and then freeze-dried. Imaging was performed using Hitachi S4800 SEM without sputter coating.

### Effect of OligoG as a treatment to inhibit biofilm formation (CLSM)

For CLSM imaging, *P. aeruginosa* NH57388A biofilms were grown in Whatman 96-well glass-bottomed plates in MH broth ± 0.5, 2, or 6% OligoG (or TxRd®-labelled OligoG). A 1:10 inoculum of the *P. aeruginosa* (NH57388A) overnight culture was used and plates were incubated at 37 °C for 24 h.

### Effect of OligoG as a treatment to disrupt established biofilms and to potentiate the effect of erythromycin and tobramycin

*P. aeruginosa* NH57388A biofilms were grown in Whatman 96-well glass-bottomed plates in MH broth using a 1:10 (v/v) inoculum of overnight culture and plates were incubated at 37 °C for 24 h prior to treatment. Half the supernatant was then carefully removed and replaced with fresh MH broth ± OligoG (or TxRd®-labelled OligoG). The final concentrations of OligoG used were 0.5, 2, and 6% (v/v). The samples were then incubated at 37 °C at each time point (1, 4, and 24 h) before imaging. For the erythromycin and tobramycin potentiation experiments, half the supernatant was removed after 24 h biofilm growth and replaced with MH broth ± 2% OligoG and 128 µg ml^−1^ (MIC value) erythromycin (final concentration; v/v) or 1 µg ml^−1^ (MIC value) tobramycin (final concentration; v/v) before a further 24 h incubation at 37 °C.

### Texas Red (TxRd^®^)-labelling of OligoG and conjugate characterisation

To follow the fate of OligoG during biofilm inhibition and disruption via CLSM, conjugation to a fluorescent dye was utilised. OligoG was labelled with TxRd® cadaverine (molecular weight 690.87 g mol^−1^) using 1-ethyl-3-[3-dimethylaminopropyl]carbodiimide hydrochloride (EDC) and N-hydroxysulfosuccinimide (sulfo-NHS) as zero-length crosslinking agents (Fig. S2a; see supplementary information for full methodology and control experiments conducted).

### Selective staining and CLSM imaging

Biofilm supernatants were carefully removed before staining. For the OligoG biofilm assays, the biofilms were stained with LIVE/DEAD® (BacLight^TM^ Bacterial Viability Kit, Molecular Probes^TM^) for 10 min, prior to imaging for bacterial visualisation. For the TxRd®-OligoG biofilm assays, the biofilms were stained with SYTO® 9 for 10 min prior to CLSM imaging. For visualisation of protein content, biofilms were stained with FilmTracer^TM^ SYPRO® Ruby Biofilm Matrix stain (Molecular Probes^TM^; 4 h incubation) or stained with TOTO-1 (Molecular Probes^TM^; 20 min incubation) in combination with Concanavalin A (ConA) Alexa Fluor 633 (Molecular Probes^TM^; 40 min incubation) for eDNA and EPS visualisation respectively. CLSM imaging of the biofilm formation and biofilm disruption assays was performed using an Olympus FV1000 CLSM. CLSM imaging of the biofilm components (SYPRO® Ruby Biofilm Matrix stain, TOTO-1, ConA) and antibiotic potentiation assays was achieved using a Leica SP5 CLSM. The CLSM images were achieved with an x63 lens (oil), a step size of 0.7 µm, line averaging of 2 and with simultaneous scanning (See supplementary information for the excitation and emissions ranges of the stains used in this study). CLSM images were processed using Imaris software (Bitplane, Concord, MA, USA) as maximum intensity images. The fluorescence intensities from the CLSM biofilm images achieved with ConA and TOTO-1 were compiled from the Imaris programme.

### COMSTAT image analysis

CLSM z-stack images were analysed using COMSTAT image analysis software for quantification of three-dimensional biofilm structures through measurement of biofilm volume, surface roughness and biofilm depth.^41^

### Semi-quantification of planktonic bacteria

The supernatant removed from the biofilms was placed into a 96-well plate and centrifuged at 3000×*g* for 10 min. The supernatant was then removed and the cell pellets re-suspended in 100 μl of PBS, after which 1 μl of crystal violet (0.5% w/v in PBS) was added into each well and the OD_595_ measured to quantify the total biomass (live and dead) of planktonic cells.

### Transwell® biofilm diffusion studies

Overnight cultures of *P. aeruginosa* NH57388A were adjusted to 10^7^ cfu ml^−1^, and 0.1 ml of culture together with 0.2 ml of MH broth were added to the 6.5 mm Transwell® (3.0 µm pore size; Corning) upper donor well, while 1 ml of MH broth was added into the lower acceptor well. The Transwell® plate was then placed on a rocker at 20 rpm at 37 °C for 24 h. After growth, the supernatant was removed from the donor well and 100 µl of MH (±0.5, 2, 6% OligoG) was added and incubated for a further 4 h. The supernatant was again removed and the biofilms rinsed twice in deionised water, before moving the donor Transwells® to a new acceptor well containing 600 µl of deionised water. Then 140 µl of 0.007% negatively-charged FluoSpheres (Carboxylate-modified Microspheres, 0.2 µm; Molecular Probes^TM^) in distilled water were placed into each donor well and after 1 h incubation, 100 µl of distilled water was removed from the acceptor well and placed into a Grenier glass-bottom 96-well black plate for fluorescence reading. A Fluostar Omega Microplate Reader was used to measure end-point fluorescence (excitation 488 nm/emission 520 nm). Readings were also taken at the 2 h incubation interval.

### Molecular dynamics (MD) simulations of calcium, DNA and OligoG CF-5/20 interactions

MD simulations were all run on the High Performance Wales Supercomputer (www.hpcwales.co.uk; Supporting Information). Sequences were generated using BIOVIA Discovery Studio® 2017 software (BVS). Alginate oligosaccharides were derived from the crystal structure of the molecule (PDB ID 1J1N4) and converted into 16 repeat units using BVS and elongated to 16 residues; correspond to the DPn of the OligoG used in the in vitro experiments. Two, 15 bp DNA sequences were generated to represent a double strand, selected from the genomes of *P. aeruginosa*;^75^ pseudomonal DNA sequences were selected to ensure their G:C ratios were representative of *P. aeruginosa* as a whole (66.6%).

### FTIR analysis of interactions of OligoG with DNA and Ca^2+^

FTIR analysis was performed on samples containing OligoG, Ca^2+^ ion solution (1000 ppm; Cole Parmer WZ-27502-59) and DNA (170 µg/ml; > 90% at 50 kB: Promega G3041). All samples were vortexed, centrifuged at 1000×*g* for 30 s, before being incubated at 37 °C for 30 min on a plate-shaker. Five µl of each sample was then pipetted onto 96-well silicon plates (Bruker Optics Inc., Billerica, MA). Following drying at room temperature for 1 h, high-throughput FTIR analysis was performed using a Bruker Vertex 70 with HTS-XT attachment and a DTGS detector (Bruker). Three replicates of each sample were analysed where infrared spectra were obtained and processed using the in-built tools and algorithms in OPUS 7.5. Spectra were vector-normalised, baseline-corrected using the automatic “rubberband” correction.

### Isothermal titration calorimetry

Buffer solutions were prepared by dissolving 5.84 g of NaCl, 4.19 g of MOPS, and 0.11 or 0.55 g of CaCl_2_ in deionised water, adjusting the pH to 7.0 (Hanna Instruments pH210 microprocessor pH metre with a VWR simple junction gel universal combined pH/reference electrode) using an aqueous solution of sodium hydroxide, and making up the solution to 1 l. OligoG solutions were prepared by dissolving 20 mg of OligoG in 1 ml of the required buffer to obtain a solution of 101 mM OligoG (concentration in terms of monomeric units). DNA stock solutions were prepared by dissolving ~ 0.1 g fish sperm (FS) DNA in 10 ml buffer. These solutions were dialysed (MWCO 3.5 kDa) overnight against half a litre of buffer and the DNA concentration was determined spectrophotometrically (JASCO V630 spectrophotometer) using an extinction coefficient of 12800 M^−1^ cm^−1^ at 260 nm.^76^ The stock solution was diluted as required to prepare 1 mM and 10 mM solutions of DNA. For all experiments, other buffer components used were 100 mM NaCl and 20 mM MOPS pH 7.0.

Calorimetric titrations were carried out at 37 °C on a MicroCal PEAQ-ITC microcalorimeter (Malvern Instruments Ltd). The instrument was operated by applying a reference power of 10 µcal/s, in high feedback mode, stirring the sample cell contents at 750 rpm, with a pre-injection initial delay of 60 s. All experiments involved an initial injection of 0.4 µl in 0.8 s followed by 12 further injections of 3.0 µl in 6.0 s into the calorimeter sample cell. Injections were spaced by at least 150 s to allow full recovery of the baseline. Raw data was treated using MicroCal PEAQ-ITC Analysis Software (1.0.0.1259) to generate integrated heat effects per injection (ΔQ). Molar heat effects per injection (ΔH) were calculated using Excel (Microsoft).

### Statistical analysis

Statistical software (Minitab v.14; Minitab, State College, PA) was used for all statistical analyses presented. Parametric data was analysed using *T*-test, while non-parametric data was analysed using Mann–Whitney test to determine significant differences for pair-wise comparisons. A *P* value of <0.05 was considered statistically significant.

### Data availability

Data generated and analysed during this study are included in this published article and its Supplemental information file. Additional details available upon reasonable request.

## Electronic supplementary material

Supplementary Information
