# Supplementary material for: Targeted disruption of the extracellular polymeric network of Pseudomonas aeruginosa biofilms by alginate oligosaccharides
Source: NPJ Biofilms Microbiomes. 2018 Jun 29;4:13. doi: 10.1038/s41522-018-0056-3 (PMC6026129; doi:10.1038/s41522-018-0056-3)
Supplement: Supplementary file 1 — Supplementary Information [file 41522_2018_56_MOESM1_ESM.pdf]

SUPPLEMENTAL MATERIAL

MATERIALS AND METHODS

Selective staining and CLSM imaging (cont.).

Table 1: Excitation/emission ranges of the stains used in this study.

Stains	Excitation (nm)	Emission (nm)
LIVE/DEAD Baclight Bacterial Viability Kit – Syto 9 (Molecular Probes^{TM})	480	500
LIVE/DEAD Baclight Bacterial Viability Kit – Propidium Iodide (Molecular Probes^{TM})	490	635
Syto 9(Molecular Probes^{TM})	485	498
Film Tracer SYPRO Ruby Biofilm Matrix (Molecular Probes^{TM})	450	610
TOTO-1 (Molecular Probes^{TM})	514	533
Concanavalin (ConA) Alexa Fluor 633 (Molecular Probes^{TM})	632	647
Texas Red used for OligoG conjugation (Molecular Probes^{TM})	590	615

Texas Red (TxRd®)-labelling of OligoG and conjugate characterisation (cont.).

Briefly, OligoG (100 mg) was dissolved in 1 ml PBS buffer (pH 7.4) in a 10 ml round-bottomed flask. To this, EDC (6.7 mg, 10 molar eq.) and sulfo-NHS (6 mg, 10 molar eq.) were added, and the mixture left stirring for 15 mins. Subsequently, TxRd® cadaverine (dissolved in dimethyl sulfoxide, DMSO; 5 mg ml^{-1} and stored at -20°C until use) was added (0.106 ml, 2 molar eq.). Then, NaOH (0.5 M) was added drop-wise to raise the pH to 8.0 and the reaction mixture was left stirring in the dark (5 h). The conjugate was purified by size exclusion chromatography (SEC) using a disposable PD-10 desalting column containing Sephadex G25 equilibrated with dH_2O (25 ml added in total). The entire reaction mixture was added to the column and 5.5 ml of the eluted dH_2O was collected, lyophilised and stored at -20°C. The reaction mixture was characterised by SEC prior to, and after purification, by analysis of SEC fractions for fluorescence (5 mg ml^{-1} solution in PBS). To estimate the proportion of free and OligoG-conjugated TxRd® was present in the conjugate, a sample of the crude reaction mixture and final product were diluted to 0.5 ml with PBS. This was added to a PD-10 column and

fractions (0.5 ml) were collected in 0.5 ml Eppendorf tubes. A sample of each fraction (100 µl) was placed in duplicate into a black 96-well microtitre plate and fluorescence measured using a fluorescent plate reader (λ_{ex} = 591 nm, λ_{em} = 612 nm, gain 1000). The values were then plotted against fraction volume and bound TxRd® was expressed as percentage of total fluorescence measure for all fractions. UV spectroscopy was used to determine the total TxRd® content of the TxRd®-OligoG conjugate. TxRd®-loading was determined by analysing a 5 mg ml^{-1} solution in PBS and relating this to a TxRd® calibration curve (1-10 µg ml^{-1}). Specific activity (µg TxRd® / mg TxRd®-labelled conjugate) was calculated using the following equation:

$$\text{Specific activity (µg TxRd/ mg TxRd conjugate)} = \frac{\text{(µg TxRd® from UV) x \% TxRd® bound}}{\text{100 x mg TxRd® -labelled conjugate}}$$

Control experiments for TxRd®-labelled OligoG. Since liberation of free TxRd® in the culture medium could potentially cause artefacts in the observed cell fluorescence (due to more rapid uptake compared to macromolecular conjugates), the levels of free TxRd® in the cell culture medium was analysed via PD-10 chromatography in specific experiments at the end of the incubation period. A sample of the culture media was added to the PD-10 column (0.5 ml) as described previously. Control experiments included addition of the equivalent concentrations of "free" TxRd® cadaverine (0.052 µg ml^{-1}, equivalent to 6% TxRd®-OligoG conjugate) to the biofilm CLSM assays.

Ability of OligoG to potentiate the effect of erythromycin and tobramycin in Minimum Inhibitory Concentration (MIC) assay. *P. aeruginosa* NH57388A was grown overnight in TSB and adjusted to 10^8 cfu ml^{-1} (~OD_{600} – 0.08; equivalent to 0.5 McFarland standard). Two-fold antibiotic serial dilutions were prepared in MH broth ± 0.5, 2, or 6% OligoG in flat-bottom 96-well microtiter plates (100µl in each well). The bacterial cultures were diluted 10-fold in MH broth, and 5 µl was added to the antibiotic serial dilutions to give

a final concentration of $5x10^5$ CFU/ml. Plates were incubated at 37°C for 18 h and MICs determined as the lowest concentration at which there was no visible growth.

Molecular dynamics (MD) simulations. MD simulations were performed using GROMACS 4.6.5 software and the AMBER03 protein and nucleic AMBER94 force-fields. Structures were boxed and solvated using the GROMACS modules 'gmx editconf' and 'gmx solvate'. The two DNA helical strands were placed centrally in a box and solvated using TIP3P (transferable intermolecular potential with 3 points). The box was approximately 236.28 nm^3 in size and filled with ~7,373 water molecules. The system was then neutralised via the addition of an appropriate concentration of calcium ions into the box. The Particle mesh Ewald method was used to analyse long-range electrostatic interactions and a 1.4 nm cut-off was applied to Lennard-Jones interactions. Initial MD simulations were performed in a two-step process. Following an energy minimization stage (EM), this was followed by MD simulation at 300 K for 100 ns allowing Ca^{2+} bridges to form between the DNA molecules. The final positions of the DNA molecules and Ca^{2+} ions were calculated and placed inside a 318.83nm^3 "box" with OligoG and filled with ~10,010 water molecules. A second round of MD simulations was performed and run over 50 ns. In a final model, the resultant positions of DNA and calcium ions from the initial models were employed and un-bound calcium ions removed from the simulation. OligoG was then added and the modelling repeated as described above.

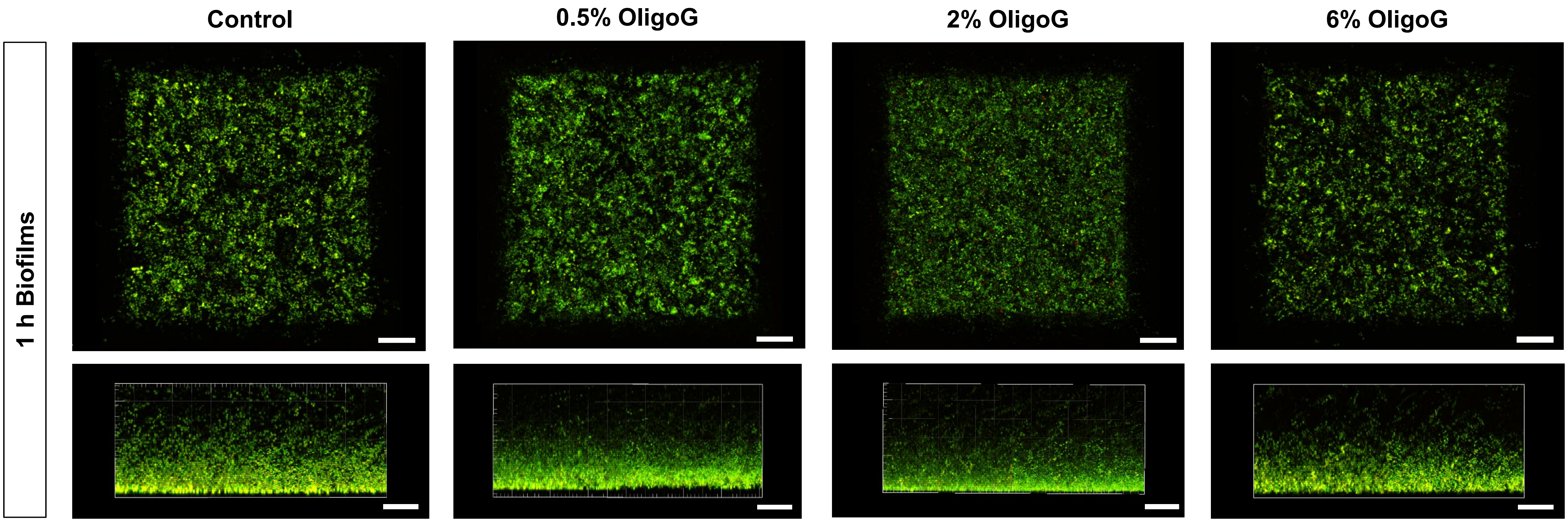

FIG S1 Effect of 1h OligoG treatment on disruption of mucoid established biofilms: CLSM 3D imaging (aerial + side view) with LIVE/DEAD® staining of *P. aeruginosa* (NH57388A) biofilms grown for 24 h at 37°C in MH broth followed by 1 h OligoG treatment (0.5%, 2% and 6%; Scale bar, 20 µm) (n=3).

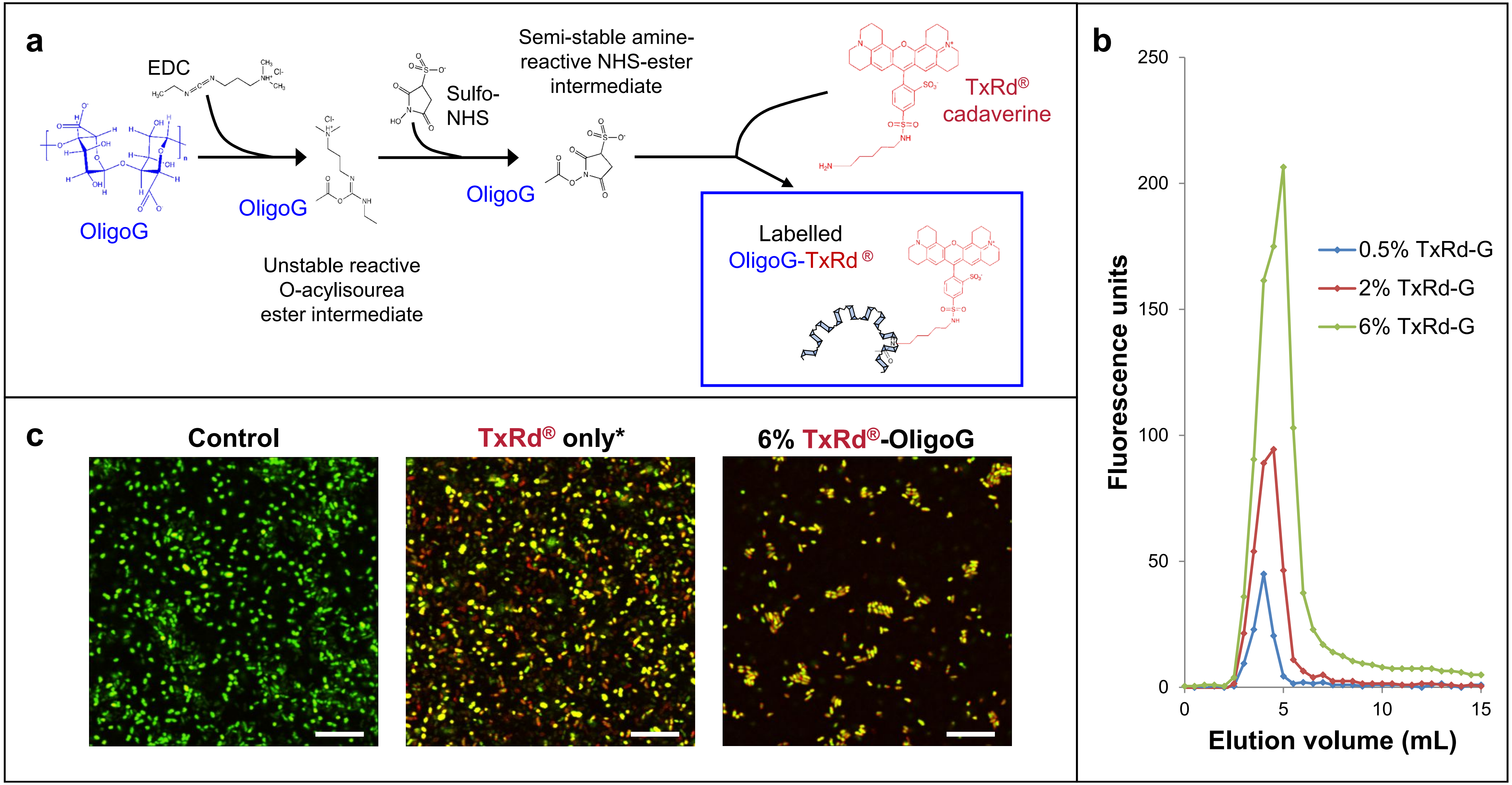

FIG S2 Texas Red (TxRd®)-labelling of OligoG and conjugate characterisation. **a** Diagrammatic summary showing TxRd® cadaverine labelling of OligoG using 1-ethyl-3-[3-dimethylaminopropyl]carbodiimide hydrochloride (EDC) and N-hydroxysulfosuccinimide (sulfo-NHS) as zero-length crosslinking agents. **b** Quantification of TxRd®-OligoG and unbound TxRd® using PD-10 column analysis of supernatant from biofilms grown for 24 h in MH broth, followed by 24 h treatment with TxRd®-OligoG (0.5%, 2% or 6%). **c** CLSM 2D imaging (a single optical section taken close to the bottom of the biofilm; scale bar, 10 µm) of *P. aeruginosa* (NH57388A) biofilms grown for 24 h in MH broth, followed by 24 h treatment with 6% TxRd®-OligoG or TxRd® only (*equivalent to 6% TxRd®-OligoG conjugate), with the respective MH only control.

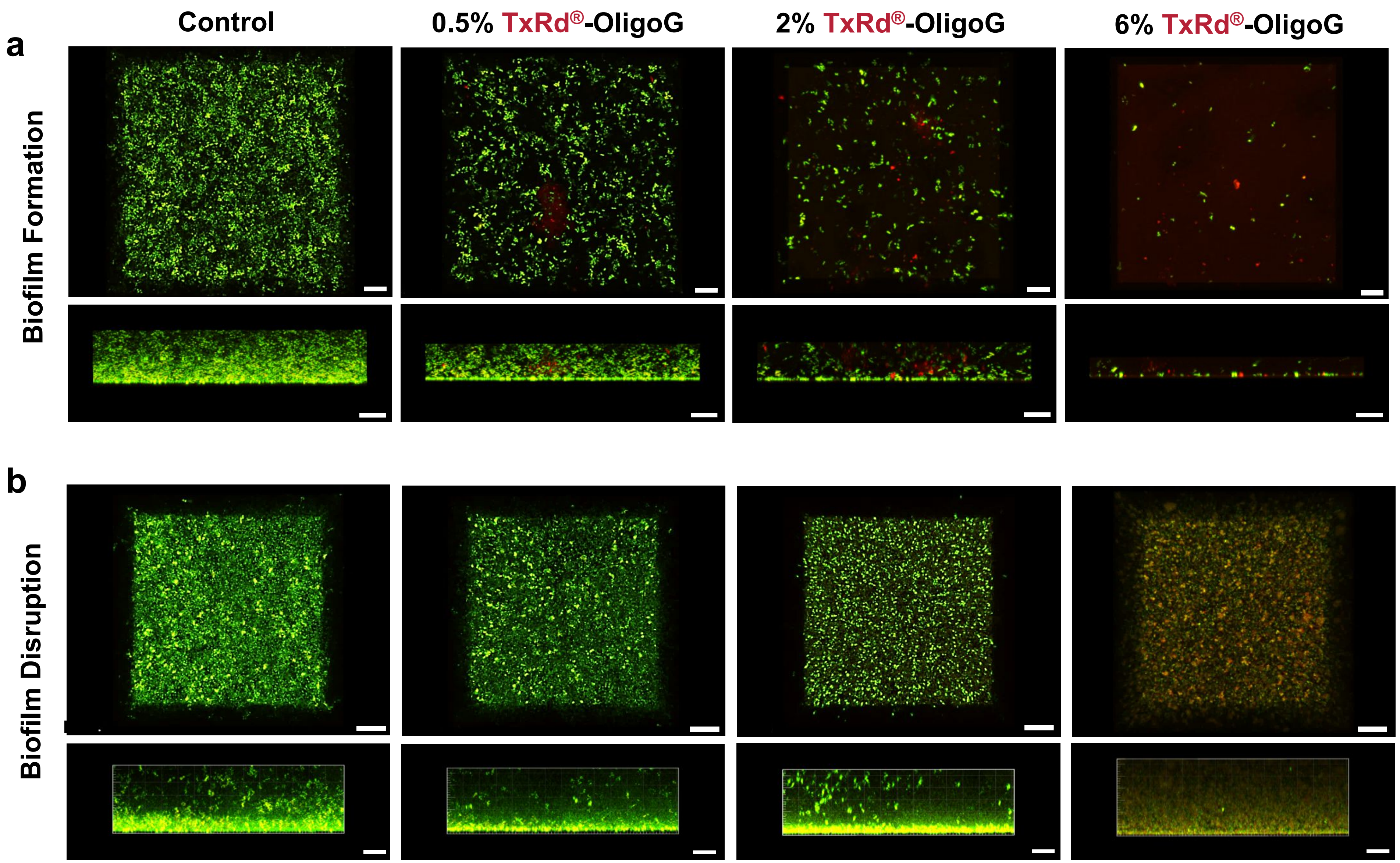

FIG S3 Characterisation of Texas Red (TxRd®)-labelled OligoG in mucoid biofilm formation and biofilm disruption assays. CLSM 3D imaging (aerial and side views) with SYTO-9® staining of *P. aeruginosa* (NH57388A) on **a** biofilm formation (biofilms grown for 24 h in MH broth ± TxRd®-OligoG, 0.5%, 2% and 6%; Scale bar, 10 µm) and **b** biofilm disruption (biofilms grown for 24 h in MH broth, followed by 24 h treatment of TxRd®-OligoG, 0.5%, 2% and 6%; scale bar, 10 µm).

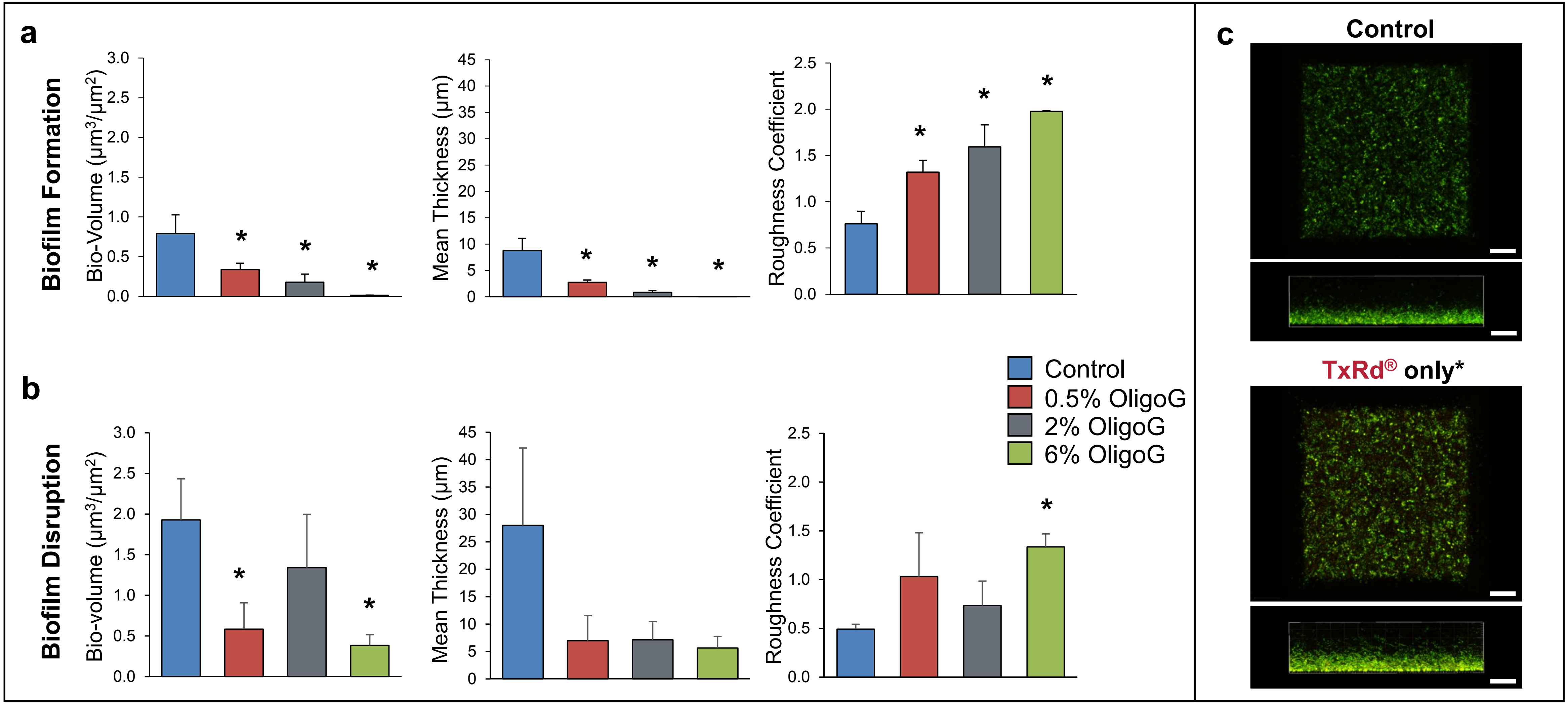

FIG S4 Quantification of the effect of Texas Red (TxRd®)-labelled OligoG in mucoid biofilm formation and biofilm disruption assays. Quantification of the effect of TxRd®-OligoG on the inhibition and disruption of mucoid *P. aeruginosa* (NH57388A) biofilms, with SYTO-9® staining (shown in Fig S3), using COMSTAT image analysis of the corresponding biofilm CLSM 3D z-stack images of **a** biofilm formation (biofilms grown for 24 h in MH broth ± TxRd®-OligoG, 0.5%, 2% and 6%) and **b** biofilm disruption (biofilms grown for 24 h in MH broth, followed by 24 h treatment of TxRd®-OligoG, 0.5%, 2% and 6%). 3D CLSM imaging showing aerial (scale bar, 20 µm) and side views (scale bar, 15 µm) of *P. aeruginosa* (NH57388A) grown for 24 h in MH broth, followed by 24 h treatment of only MH broth control or TxRd® control 0.052 µg/ml [*equivalent to 6% TxRd®-OligoG conjugate] imaged with SYTO® 9 staining. *P <0.05 was determined by comparison to the untreated control. Error bars represent the standard deviation of the data set (n=3).

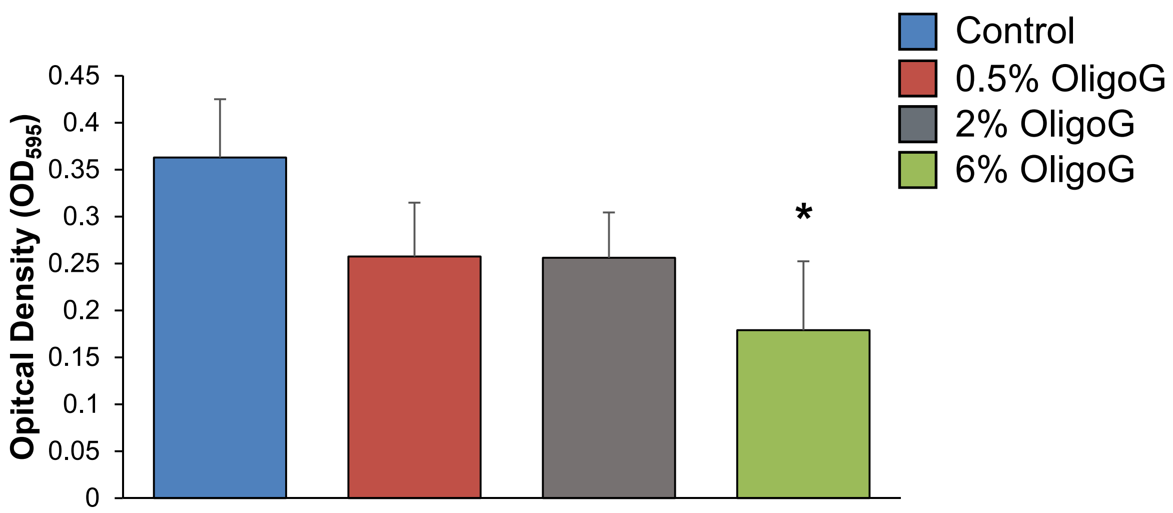

FIG S5 Effect of OligoG on the release of planktonic cells from disrupted mucoid established biofilms. Quantitative analysis of planktonic bacterial cells using crystal violet staining of *P. aeruginosa* (NH 57388A) biofilm supernatants from established biofilms (biofilm disruption assay) grown at 37ºC in MH broth for 24h, followed by 24 h treatment of ± OligoG (0.5%, 2% and 6%) **P* <0.05 significance was determined by comparison to the untreated control. Error bars represent the standard deviation of the data set (n=3).

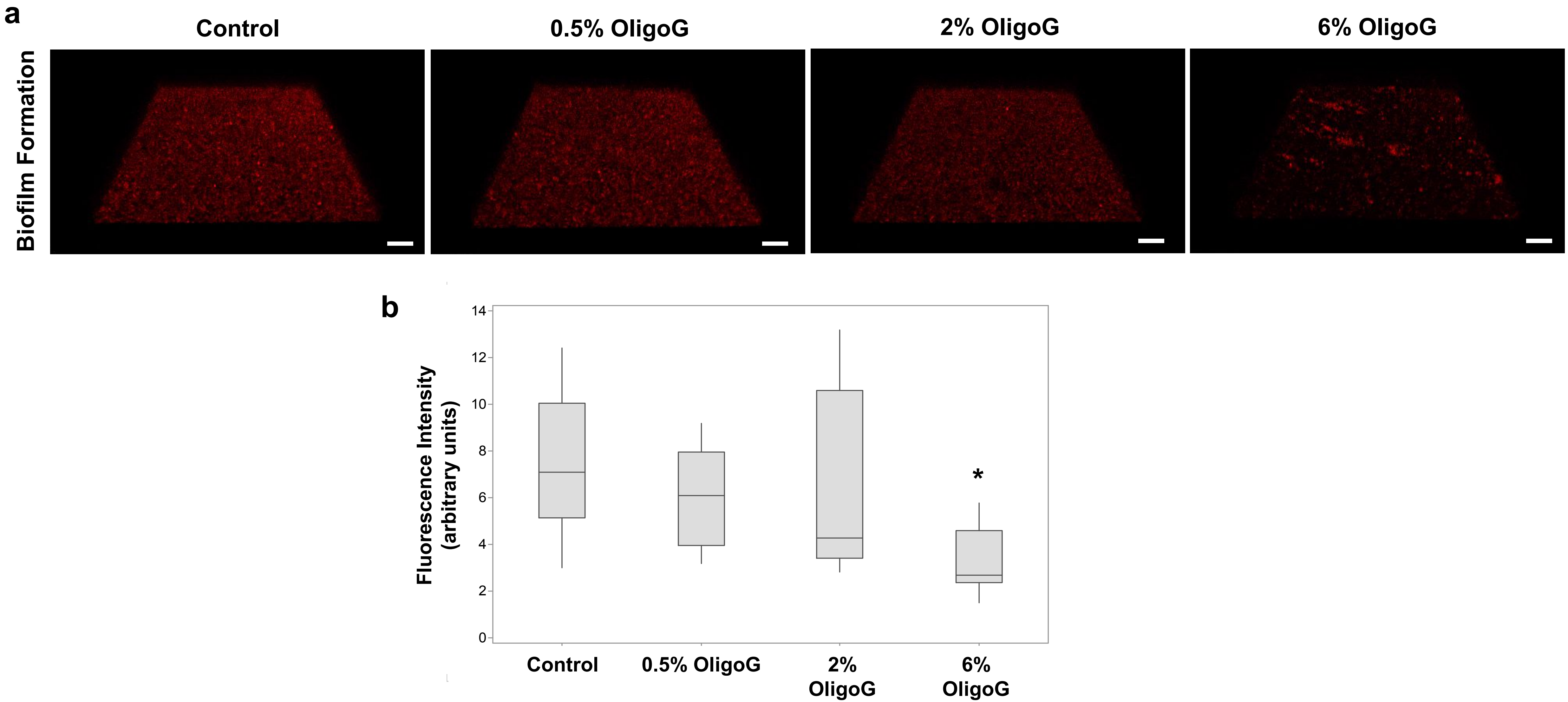

FIG S6 Effect of OligoG on the protein component of mucoid biofilms. **a** CLSM 3D imaging of *P. aeruginosa* (NH57388A) biofilms grown for 24 h in MH broth ± OligoG (0.5%, 2% and 6%), using SYPRO Ruby Biofilm matrix® staining (for protein visualisation, red; scale bar, 30 µm). **b** Corresponding mean fluorescence intensities (arbitrary units x10^6) achieved from CLSM 3D imaging of the biofilm formation assay. *P <0.05 significance was determined by comparison to the untreated control. Error bars represent the standard deviation of the data set (n=3).

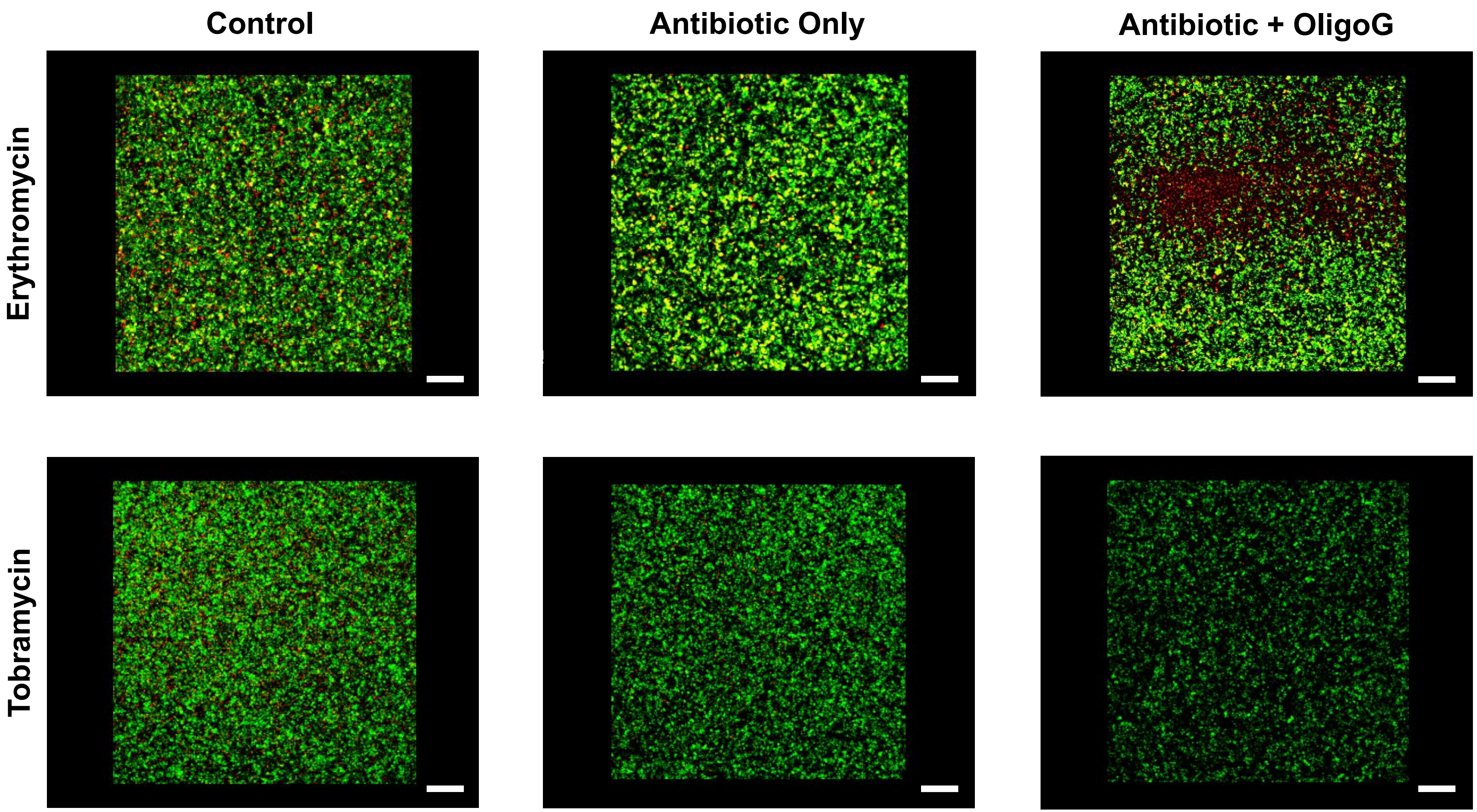

FIG S7. Ability of OligoG to potentiate the effect of erythromycin and tobramycin against mucoid established biofilms. CLSM 3D imaging (aerial view) with LIVE/DEAD® staining of *P. aeruginosa* (NH57388A) biofilms grown for 24 h at 37°C in MH broth followed by 24 h treatment with OligoG (2%) and/or erythromycin (128 µg ml^{-1} =MIC) and tobramycin (1 µg ml^{-1} =MIC; scale bar, 30 µm).

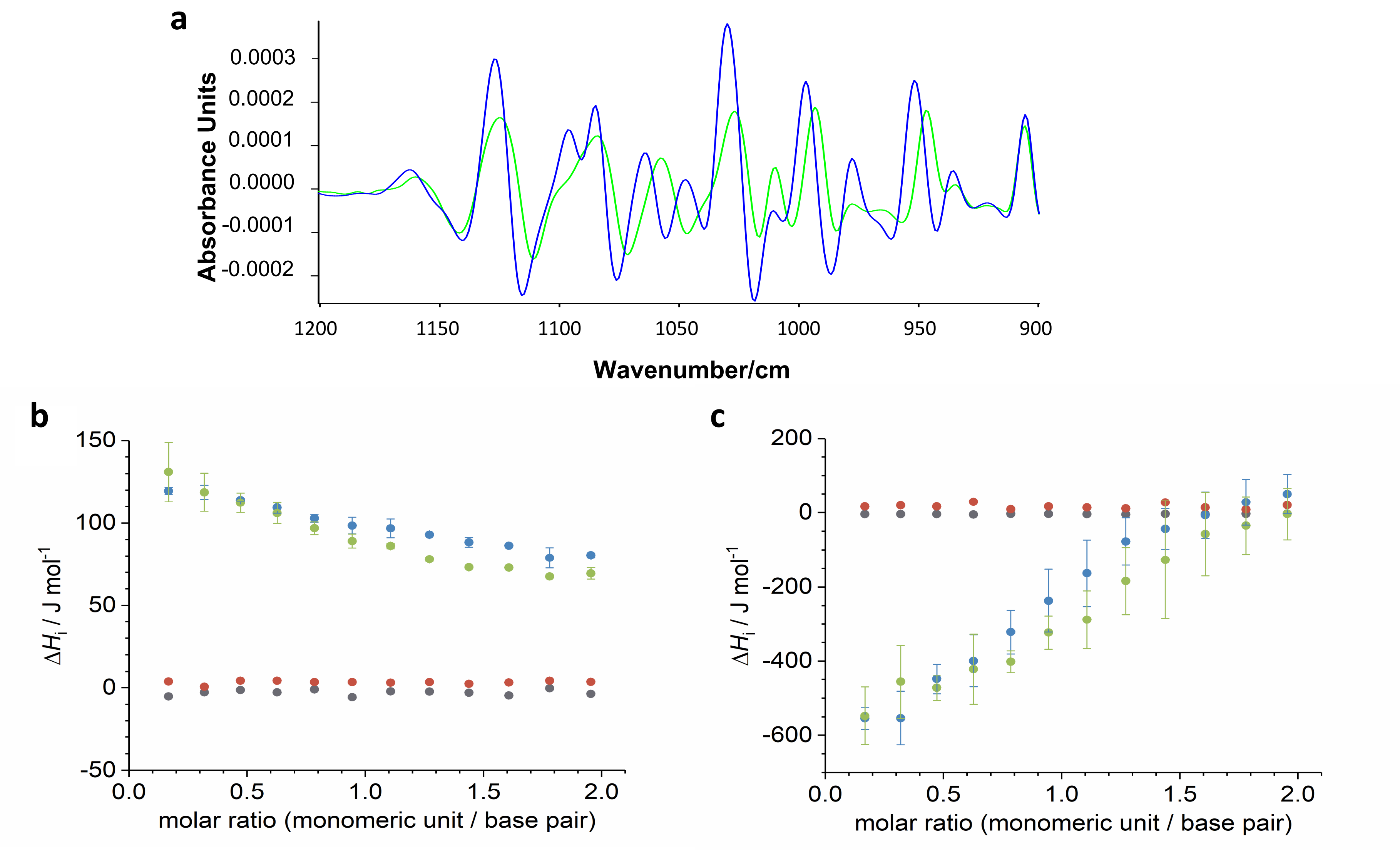

FIG S8 FTIR and ITC analysis of interactions of OligoG with DNA and Ca^{2+}. **a** FTIR Inverted (peaks are pointing upwards) second derivative spectra of 1200-900 cm^{-1} with pure OligoG (green) and OligoG + 5mM Ca^{2+} (red). Isothermic calorimetric titrations of 101 mM OligoG into 10 mM FS DNA containing **b** 1 mM $CaCl_2$ or **c** 5 mM $CaCl_2$ (and corresponding reference experiments); buffer into buffer (grey), buffer into DNA (red), OligoG into buffer (blue), OligoG into FS DNA (green). Concentrations of OligoG and FS DNA are in terms of monomeric units and base pairs, respectively. Error bars represent the standard deviation of the data set (n=3).
